# Striatal Neurons Expressing D_1_ and D_2_ Receptors are Morphologically Distinct and Differently Affected by Dopamine Denervation in Mice

**DOI:** 10.1038/srep41432

**Published:** 2017-01-27

**Authors:** D. Gagnon, S. Petryszyn, M. G. Sanchez, C. Bories, J. M. Beaulieu, Y. De Koninck, A. Parent, M. Parent

**Affiliations:** 1 Centre de recherche de l’Institut universitaire en santé mentale de Québec, Department of Psychiatry and Neuroscience, Faculty of medicine, Université Laval, Quebec City, QC, Canada

## Abstract

The loss of nigrostriatal dopamine neurons in Parkinson’s disease induces a reduction in the number of dendritic spines on medium spiny neurons (MSNs) of the striatum expressing D_1_ or D_2_ dopamine receptor. Consequences on MSNs expressing both receptors (D_1_/D_2_ MSNs) are currently unknown. We looked for changes induced by dopamine denervation in the density, regional distribution and morphological features of D_1_/D_2_ MSNs, by comparing 6-OHDA-lesioned double BAC transgenic mice (*Drd1a*-tdTomato/*Drd2*-EGFP) to sham-lesioned animals. D_1_/D_2_ MSNs are uniformly distributed throughout the dorsal striatum (1.9% of MSNs). In contrast, they are heterogeneously distributed and more numerous in the ventral striatum (14.6% in the shell and 7.3% in the core). Compared to D_1_ and D_2_ MSNs, D_1_/D_2_ MSNs are endowed with a smaller cell body and a less profusely arborized dendritic tree with less dendritic spines. The dendritic spine density of D_1_/D_2_ MSNs, but also of D_1_ and D_2_ MSNs, is significantly reduced in 6-OHDA-lesioned mice. In contrast to D_1_ and D_2_ MSNs, the extent of dendritic arborization of D_1_/D_2_ MSNs appears unaltered in 6-OHDA-lesioned mice. Our data indicate that D_1_/D_2_ MSNs in the mouse striatum form a distinct neuronal population that is affected differently by dopamine deafferentation that characterizes Parkinson’s disease.

The striatum is the main input structure as well as the largest integrative component of the basal ganglia. It receives a multitude of neurochemical inputs that are largely processed by striatal projection neurons. At the somatodendritic level, these cells form a rather morphologically homogeneous population, each element being endowed with a medium-sized cell body and typical spiny dendrites. These so-called medium spiny neurons (MSNs) all use γ-aminobutyric acid (GABA) as a neurotransmitter and represent approximately 90–95% of the striatal neuronal population in rodents^1^. Despite their morphological similarities, MSNs can be divided into two subpopulations based on their neurochemical content and axonal projection sites. Roughly half of the MSNs express dopamine (DA) receptor of the D_1_ type and contain the neuropeptides substance P (SP) and dynorphin (DYN). They innervate mainly the substantia nigra *pars reticulata* and the entopeduncular nucleus (rodent homologue of primate internal pallidum) and form the so-called “direct pathway”. The other half of the MSNs expresses DA receptor of the D_2_ type and contains the neuropeptide enkephalin (ENK). Their axon arborizes principally in the pallidum (rodent homologue of primate external pallidum) and forms the first segment of the so-called “indirect pathway”^2^,^3^,^4^,^5^. However, it is worth noting that single-axon tracing experiments in rodents^6^ and primates^7^ indicate that most striatofugal axons arborize into the three main striatal targets. To this regards, it has recently been shown that both D_1_ and D_2_ MSNs located in the nucleus accumbens (Acb) can either inhibit or disinhibit thalamic activity depending on their projection pattern and not on their genetic characteristics^8^.

The D_1_ and D_2_ receptors are reportedly co-expressed in a certain proportion of MSNs, but the size of such D_1_/D_2_ subpopulation is still a matter of controversy. Earlier studies undertaken with *in situ* hybridization methods, immunohistochemistry or reverse transcription polymerase chain reaction have reported high percentages of striatal neurons expressing both DA receptors^3^,^9^,^10^,^11^,^12^,^13^,^14^,^15^,^16^,^17^, but a much smaller number of D_1_/D_2_ MSNs was detected in transgenic mice expressing fluorescent reporters for D_1_ or D_2_^18^,^19^,^20^,^21^.

When co-expressed by MSNs, the D_1_ and the D_2_ receptors are reportedly able to form heteromers, the activation of which can lead to a distinct intracellular signalling pathway^22^,^23^,^24^,^25^,^26^. The independent activation of the D_1_ or the D_2_ DA receptor is known to differentially regulate cyclic-AMP activity, respectively leading to the activation (D_1_ coupled to G_s_) or inhibition (D_2_ coupled to G_i_) of MSNs^27^,^28^. In contrast, the activation of D_1_/D_2_ heteromers would result in a distinct phospholipase C-mediated calcium signalling through activation of G_q_ protein^22^,^23^,^24^,^25^. However, while co-expression of D_1_ and D_2_ receptors is well accepted, the existence of D_1_/D_2_ heteromers *in vivo* remains controversial^29^,^30^.

The fate of MSNs expressing D_1_ or D_2_ DA receptor in the context of DA striatal deafferentation that characterizes Parkinson’s disease (PD) has already been studied. In a mouse model of PD, lesion of the striatal DA input was shown to induce spine pruning, principally on the D_2_ MSNs^31^,^32^ and less markedly on the D_1_ MSNs^31^,^32^,^33^,^34^,^35^. Such spine pruning appears to be a highly plastic phenomenon since the reduction of spine density observed on the D_2_ MSNs, but not that on D_1_ MSNs, could be restored by long-term administration of L-Dopa^33^,^35^. Reduction in the number of dendritic spine of striatal MSNs has also been reported in non-human primate model of PD^36^ and in PD patients^37^,^38^,^39^. Surprisingly, the fate of MSNs expressing both the D_1_ and the D_2_ DA receptors (the D_1_/D_2_ MSNs) has never been investigated in PD condition. Therefore, we have designed a study to provide the first detailed description of changes induced by DA denervation in the density, regional distribution and fine morphological characteristics of dendritic processes of D_1_/D_2_ MSNs that populate the dorsal striatum and the Acb, the main component of the ventral striatum, of mice. Using stereological approaches and single-neuronal injections performed on striatal sections from sham and 6-hydroxydopamine (6-OHDA)-lesioned double BAC transgenic mice (*Drd1a*-tdTomato/*Drd2*-EGFP), we show that the D_1_/D_2_ MSNs are affected differently than the D_1_ and D_2_ MSNs by striatal DA deafferentation that characterizes PD.

## Results

### Unilateral 6-OHDA injections cause severe TH+ cell loss in the SNc and VTA, significant DA depletion in the striatum and in the Acb and spontaneous ipsilateral rotations

**Figure 1:**
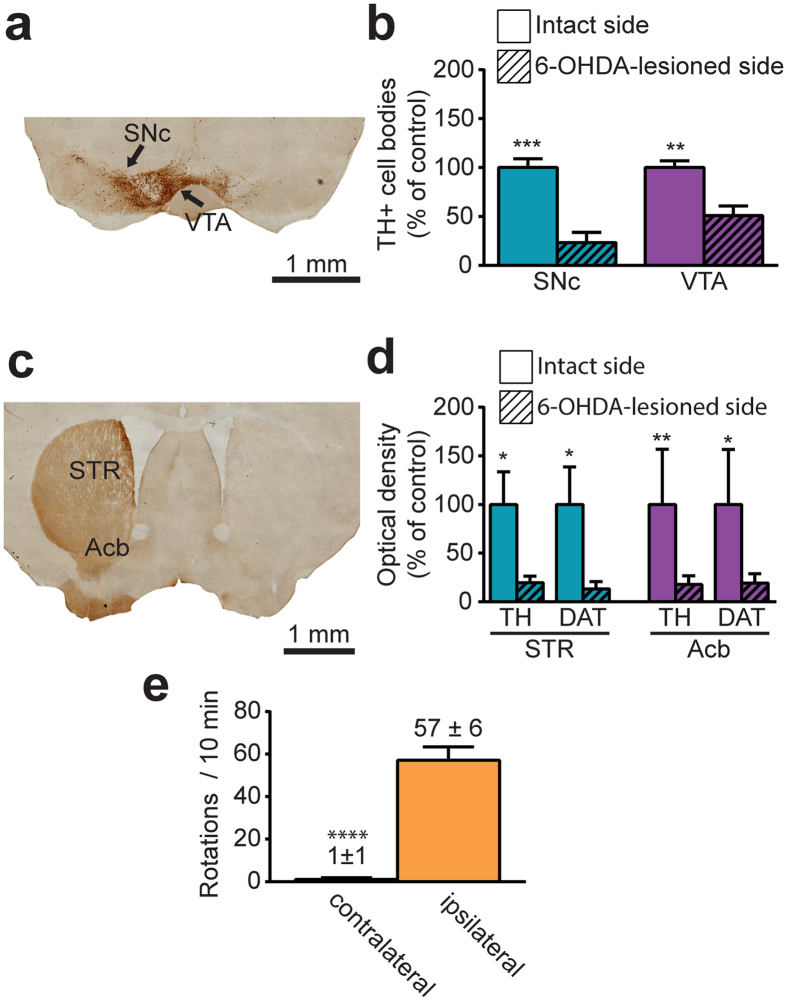
Assessment of the dopaminergic lesion induced by 6-OHDA injection in the medial forebrain bundle. (**a)** Transverse section taken through the substantia nigra *pars compacta* (SNc) and the ventral tegmental area (VTA) that was immunostained for tyrosine hydroxylase (TH) to assess the dopaminergic lesion induced by stereotaxic injection of 6-OHDA in the right medial forebrain bundle. (**b**) Histogram showing the percentage of TH + cell loss in the SNc and the VTA, as expressed in percentage of intact side. (**c**) Transverse section taken through the striatum (STR) and immunostained for TH. (**d**) Histogram showing immunoreactivity of the STR and the nucleus accumbens (Acb) for the tyrosine hydroxylase (TH) and the dopamine transporter (DAT) in the 6-OHDA-lesioned side, as expressed in percentage of intact side. (**e**) Behavioural response induced by 6-OHDA lesion, as shown in number of contralateral and ipsilateral spontaneous rotations observed in 10 minutes. *P < 0.05, **P < 0.01, ***P < 0.001 for intact side vs. 6-OHDA-lesioned side, ****P < 0.0001 for ipsilateral vs. contralateral rotations, by Mann-Whitney test.

Immunolabeling of the striatum and the substantia nigra *pars compacta* (SNc) for the DA transporter (DAT) and tyrosine hydroxylase (TH) indicate a severe DA lesion caused by 6-OHDA unilateral injections performed in the medial forebrain bundle (Fig. 1). Counts of TH + cell bodies in the midbrain show a more severe DA cell loss in the SNc compared to the VTA (80.7 ± 7.5% decrease in SNc vs. 48.9 ± 9.2% decrease in VTA, compared to control side, Fig. 1a,b). LI-COR® slide scanner measurements indicate an average of 81.3% decrease of TH and 87.5% of DAT immunoreactivity in the dorsal striatum when compared to control side as well as a 80.1% reduction of TH and a 79.3% decrease of DAT immunoreactivity in the Acb, when compared to control side (Fig. 1c,d). Behavioral assessments show a significant preference for spontaneous rotations ipsilateral to the lesioned side in mice that were unilaterally injected with 6-OHDA (57.1 ± 6.4 spontaneous ipsilateral rotations/10 min vs. 1.1 ± 0. 8 contralateral rotations, *P* < 0.0001, Fig. 1e).

### The D_1_/D_2_ MSNs contain dynorphin but not enkephalin

**Figure 2:**
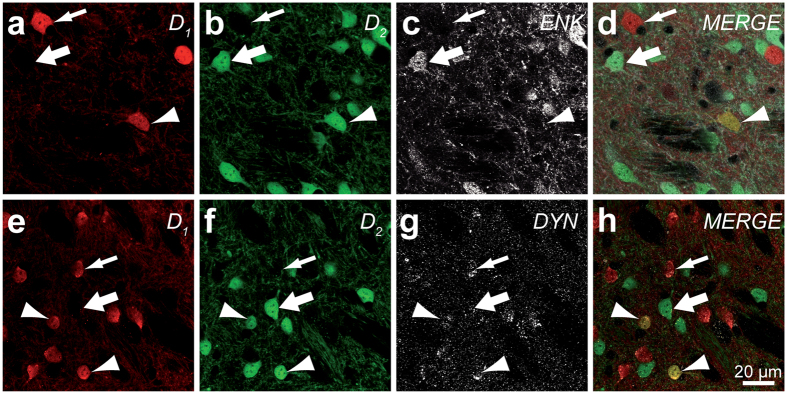
Neurochemical content of the D_1_/D_2_ MSNs. Transverse sections taken from the dorsolateral striatum of a D_1_/D_2_ transgenic mouse that were immunostained for enkephalin (ENK, **a–d**) or dynorphin (DYN, **e–h**). Thin arrows indicate D_1_ MSNs, thick arrows point to D_2_ MSNs and arrowheads to D_1_/D_2_ MSNs. The D_1_/D_2_ MSNs are immunoreactive for dynorphin but not for enkephalin in the D_1_/D_2_ transgenic mouse.

Examination of ENK and DYN-immunostained sections from colchicine-treated mice reveals that, as expected, D_1_ MSNs are immunoreactive for DYN but not for ENK. In contrast, D_2_ MSNs contain ENK but are devoid of DYN. In regard to D_1_/D_2_ MSNs, they are immunopositive for DYN but immunonegative for ENK (Fig. 2).

### D_1_/D_2_ MSNs are homogeneously distributed throughout the dorsal striatum but heterogeneously scattered in the nucleus accumbens

**Figure 3:**
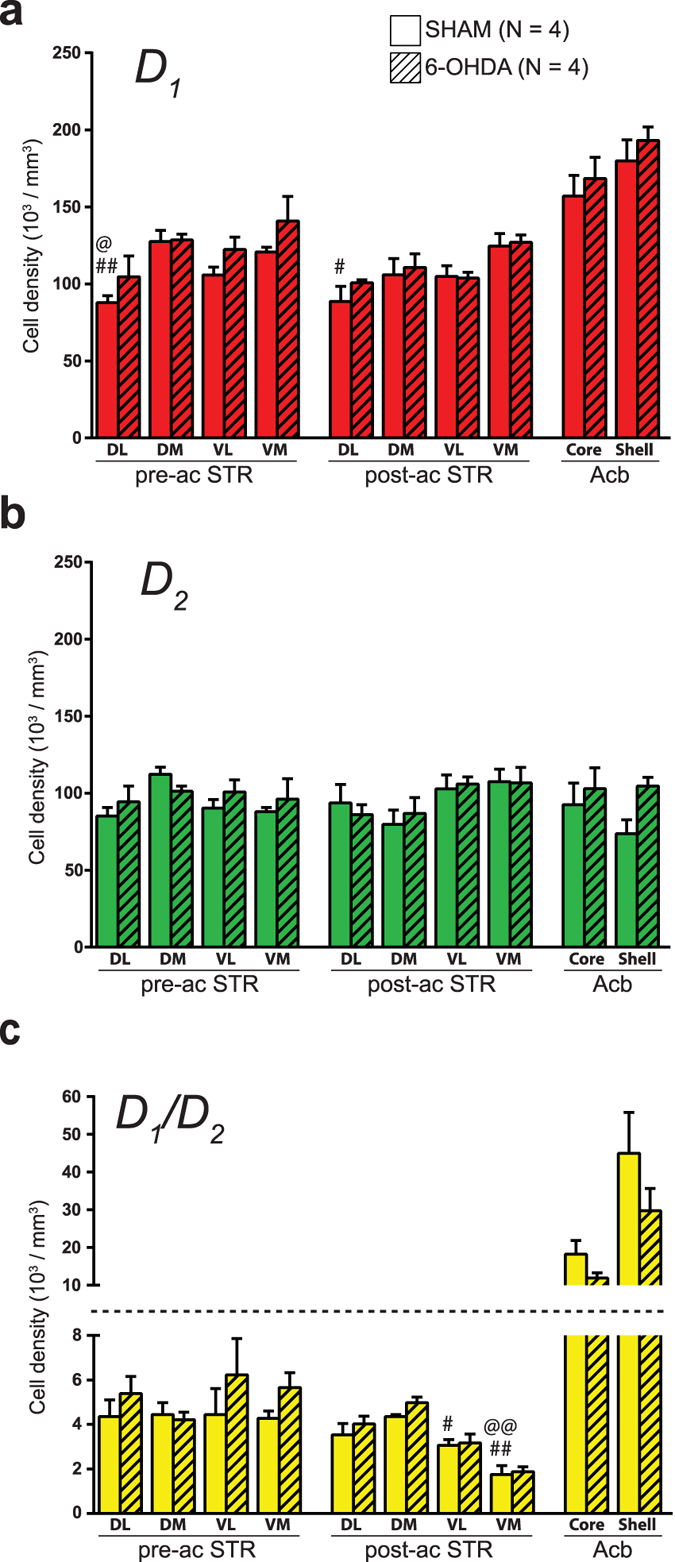
Densities of D_1_, D_2_ and D_1_/D_2_ MSNs in sham and 6-OHDA-lesioned mice. Histograms showing the density of D_1_ (**a**), D_2_ (**b**) and D_1_/D_2_ (**c**) MSNs in different regions of the striatum (STR) and the nucleus accumbens (Acb) of sham and 6-OHDA-lesioned mice. ^#^*P* < 0.05, ^##^*P* < 0.01 vs. the shell compartment of the Acb and ^@^*P* < 0.05, ^@@^*P* < 0.01 vs. the core compartment of the Acb, by Kruskal-Wallis test.

**Figure 4:**
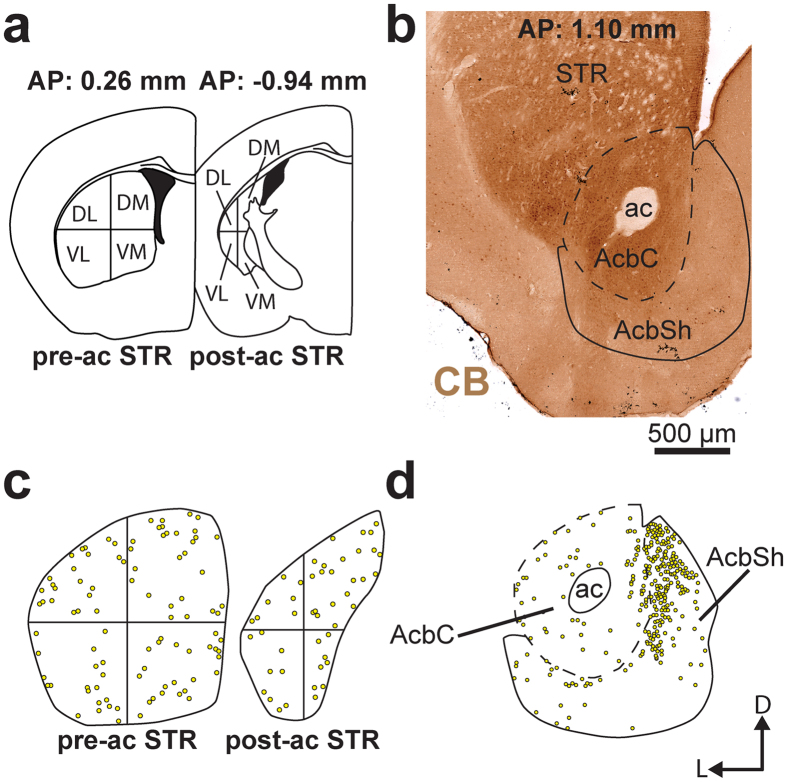
Regional distribution of D_1_/D_2_ MSNs in the dorsal striatum and the nucleus accumbens. **(a**,**b)** Schematic representations of transverse sections taken at 0.26, −0.94 and 1.10 mm from bregma on which sectors that were sampled to provide unbiased stereological estimation of the number of D_1_, D_2_ and D_1_/D_2_ MSNs in the striatum (STR, **a**) and the nucleus accumbens (Acb, (**b**) are delineated. (**c,d)** Schematic representations of the distribution of D_1_/D_2_ MSNs at the pre and post-commissural level of the STR (**c**) as well as in the core (AcbC) and the shell (AcbSh) of the nucleus accumbens. The transverse section shown in (**b**) was immunostained for calbindin and used to delineate the AcbC from the AcbSh.

The regional distribution of D_1_, D_2_ and D_1_/D_2_ MSNs in the dorsal striatum of sham mice was estimated stereologically. The overall densities of D_1_ and D_2_ MSNs are 108 ± 5 and 95 ± 4 × 10^3^ cells/mm^3^, representing respectively 52.2 ± 1.1% and 45.9 ± 1.1% of the total MSNs population of the striatum. In contrast, the D_1_/D_2_ MSNs have a much lower density with only 3.8 ± 0.3 × 10^3^ cells/mm^3^ (*P* < 0.0001), representing 1.9 ± 0.2% of the striatal MSNs. Although not statistically significant (*P* = 0.2341), the density of the D_1_/D_2_ MSNs in the ventromedial sector of the striatum was lower in the post-commissural striatum (1.8 ± 0.4 × 10^3^ cells/mm^3^) than in the pre-commissural striatum (4.3 ± 0.3 × 10^3^ cells/mm^3^). Statistical evaluations (ANOVA) of neuronal densities in different striatal regions reveal no statistical differences (Fig. 3), supporting the homogeneous regional distribution of the D_1_, D_2_ and D_1_/D_2_ MSNs throughout the dorsal striatum. Occasionally, some D_1_/D_2_ MSNs can be seen to form small clusters of 2–3 cells at different striatal levels. Assessment of the distribution of D_1_/D_2_ MSNs in striosomes and matrix striatal compartments, as delineated on immunostained sections for μ-opioid receptor, reveals no significant difference, neither at the pre-commissural level (*P* = 0.4857), nor at the post-commissural level (*P* = 0.8286, Fig. S1). Our quantitative analyses reveal that the D_1_/D_2_ MSNs are more heterogeneously distributed in the Acb than in the dorsal striatum. As shown in Fig. 4d, a dense region located in the medial part of the shell of the Acb was almost entirely composed of the D_1_/D_2_ type of MSNs. A closer examination of the lateral striatal stripe indicates no significant difference regarding the density of the D_1_/D_2_ MSNs compared to other regions of the lateral area of the shell of the Acb. The density of the D_1_/D_2_ MSNs is overall significantly higher in the Acb compared to the striatum. This difference becomes statistically significant in the shell compartment of the Acb with 45.0 ± 10.9 × 10^3^ cells/mm^3^ compared to 3.8 ± 0.3 × 10^3^ cells/mm^3^ in the dorsal striatum (*P* = 0.0098, Fig. 3c). Our stereological estimations indicate that the D_1_/D_2_ MSNs represent 14.6 ± 3.0% of the MSN population in the shell and 7.3 ± 2.2% in the core compartment of the Acb, percentages that are significantly higher than what was noted in the dorsal striatum (1.9 ± 0.2%, *P* = 0.0098). Interestingly, the density of the D_1_ MSNs is also higher in the shell of the Acb when compared to the dorsal striatum (180.0 ± 13.6 vs. 108.2 ± 5.4 × 10^3^ cells/mm^3^, *P* = 0.0244, Fig. 3a) whereas no difference is noted regarding the density of the D_2_ MSNs between the dorsal striatum and the Acb. A higher density of the D_1_ and the D_1_/D_2_ MSNs in the Acb is congruent with an overall higher density of all MSNs in the shell (298.6 ± 15.0) and the core (267.9 ± 24.5 cells/mm^3^) compartments of the Acb compared to the dorsal striatum (123.5 ± 5.4 × 10^3^ cells/mm^3^).

### The striatal D_1_/D_2_ MSNs display a smaller cell body and a shorter dendritic arborization than the two other types of MSNs

**Figure 5:**
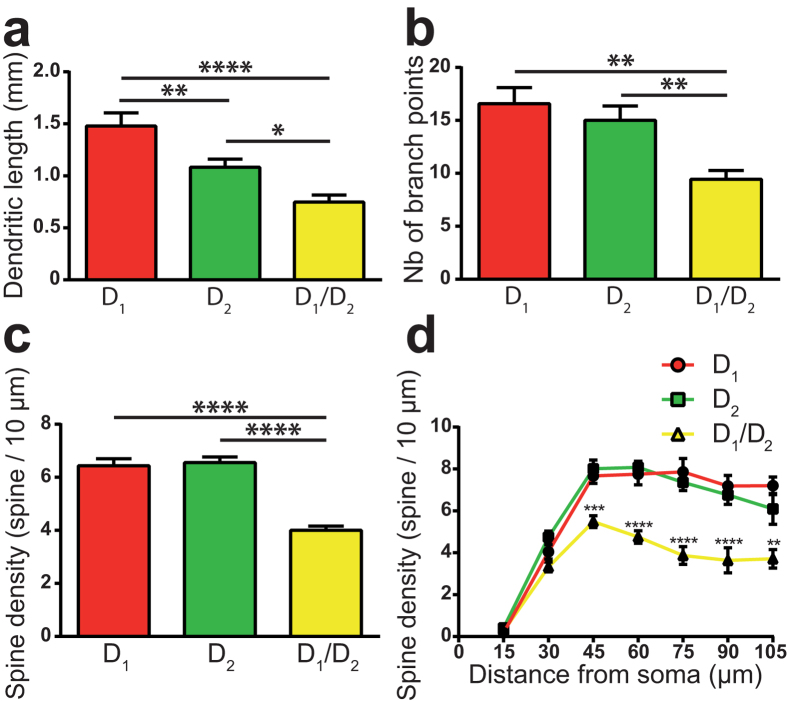
Dendritic domains of the D_1_, D_2_ and D_1_/D_2_ MSNs in sham-lesioned mice . **(a–c)** Histograms showing the total dendritic length (**a**), the number of dendritic branch points (**b**) and the overall spine density (**c**) of the D_1_ (red), the D_2_ (green) and the D_1_/D_2_ (yellow) striatal MSNs in sham-lesioned mice. (**d)** Sholl analysis of spine density of the 3 types of MSNs, as measured in sham-lesioned mice. *P < 0.05, **P < 0.01, ***P < 0.001, ****P < 0.0001 for D_1_ vs. D_2_ vs. D_1_/D_2_ by One-way (**a–c**) or Two-way (**d**) ANOVA, followed by Bonferroni’s multiple comparison test.

In sham animals, the cell body of dorsal striatum D_1_/D_2_ MSNs injected with Lucifer yellow are smaller than those of the D_1_ and the D_2_ MSNs: the mean diameter of the D_1_/D_2_ MSN perikarya is 12.5 ± 0.4 μm compared to 14.3 ± 0.4 μm for the D_1_ (*P* = 0.0094) and to 15.0 ± 0.5 μm for the D_2_ MSNs (*P* = 0.0009). The reconstruction of somatodendritic domains of Lucifer yellow-filled MSNs reveals that the total dendritic length of D_1_/D_2_ MSNs is also smaller than that of the D_1_ and the D_2_ MSNs. The mean total dendritic length for the D_1_/D_2_ MSNs is 0.75 ± 0.06 mm compared to 1.48 ± 0.13 mm for D_1_ (*P* < 0.0001) and 1.08 ± 0.08 mm for D_2_ MSNs (*P* = 0.0296, Fig. 5a). We also noted that the dendritic arborization of the D_1_ MSNs is significantly longer than that of the D_2_ MSNs (*P* = 0.0089). The dendritic arborization of the D_1_/D_2_ MSNs is significantly less profuse than that of the D_1_ and D_2_ MSNs, as indicated by a smaller number of dendritic branching points (9.5 ± 0.8 branching points) compared to the D_1_ (16.6 ± 1.5 branching points, *P* = 0.0015) and the D_2_ MSNs (15.0 ± 1.4 branching points, *P* = 0.0087, Fig. 5b).

### D_1_/D_2_ MSN dendrites harbor fewer spines than the two other types of MSNs

By dividing the number of spines by the total dendritic length for each reconstructed neuron in sham animals, we were able to evaluate the overall spine density for each of the three types of striatal MSNs. Our data reveal that the D_1_/D_2_ MSNs have a 37% lower spine density (4.0 ± 0.2 spines/10 μm) than the D_1_ (6.4 ± 0.3 spines/10 μm, *P* < 0.0001) and the D_2_ (6.6 ± 0.2 spines/10 μm, *P* < 0.0001) MSNs (Fig. 5c). A Sholl analysis performed on all reconstructed neurons indicates that this lower spine density is maintained throughout the entire dendritic extent of the D_1_/D_2_ MSNs, the difference being statistically significant on a distance ranging between 45 and 105 μm from their parent cell bodies (Fig. 5d).

### The density of D_1_/D_2_ MSNs is unaltered in 6-OHDA-lesioned mice

In 6-OHDA mice, the density of the D_1_/D_2_ MSNs in the dorsal striatum was 3.8 ± 0.3 × 10^3^ cells/mm^3^, accounting for 2.1 ± 0.2% of total MSN population. In comparison, the density of the D_1_ and the D_2_ MSNs was 108.2 ± 5.4 × 10^3^ cells/mm^3^ and 94.9 ± 4.1 × 10^3^ cells/mm^3^, representing 53.5 ± 1.0% and 44.4 ± 1.1% of the total MSN population, respectively (Fig. 3). There is no statistically significant difference between sham and 6-OHDA-lesioned animals in regard to these figures. Likewise, assessment of MSN densities in the Acb does not reveal any significant differences between the sham and 6-OHDA-lesioned animals (Fig. 3). The density of the D_1_/D_2_ MSNs obtained in the shell compartment of the Acb of 6-OHDA-lesioned mice was 29.7 ± 6.0 × 10^3^ cells/mm^3^ whereas the neuronal density of D_1_ and D_2_ MSNs in the same experimental group were 193.1 ± 8.7 and 104.7 ± 5.7 × 10^3^ cells/mm^3^, respectively. Values obtained in the core compartment of the Acb of 6-OHDA-lesioned mice reach 11.9 ± 1.4, 168.4 ± 13.8 and 102.9 ± 13.6 × 10^3^ cells/mm^3^ for the D_1_/D_2_, D_1_ and D_2_ MSNs, respectively (Fig. 3).

### The extent of D_1_/D_2_ MSN dendritic arborization is unaffected by 6-OHDA lesion, in contrast to that of D_1_ and D_2_ MSNs

**Figure 6:**
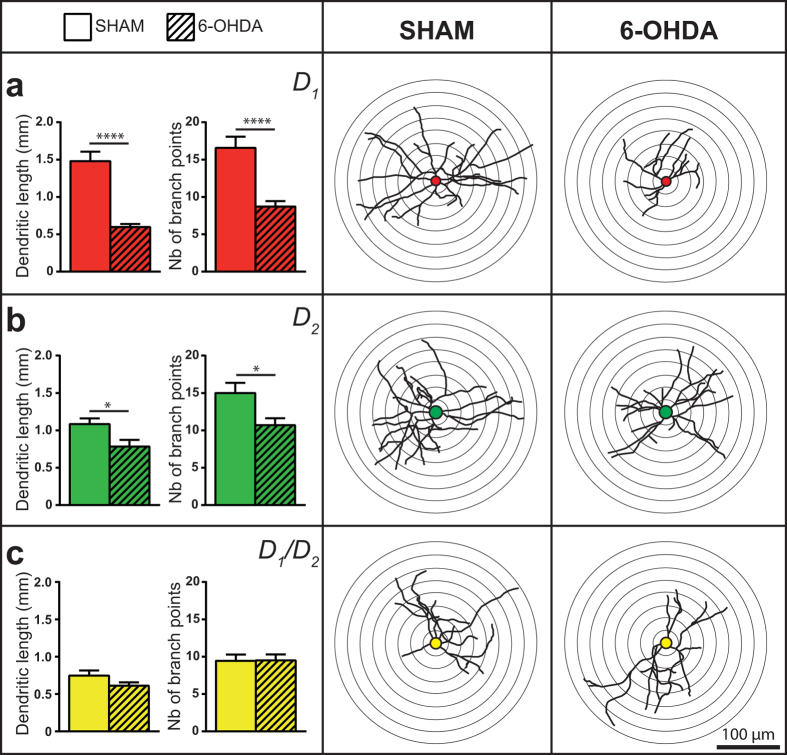
Dendritic arborization of the D_1_, D_2_ and D_1_/D_2_ MSNs in 6-OHDA-lesioned mice. Histograms showing the total dendritic length and the number of dendritic branch points in sham (plain columns) and 6-OHDA (hatched columns) lesioned mice. The center and right columns provide schematic representations of D_1_ (red), D_2_ (green) and D_1_/D_2_ (yellow) MSNs dendritic arborization in sham (center column) and 6-OHDA (right column) lesioned mice. *P < 0.05, ****P < 0.0001 for sham vs. 6-OHDA-lesioned mice, by Student’s T-test.

Statistical comparison between sham and 6-OHDA-lesioned mice in regard to the extent of somatodendritic domain belonging to the D_1_, D_2_ and D_1_/D_2_ MSNs in the dorsal striatum indicates that the total dendritic length of the D_1_ MSNs was reduced in the DA-depleted striatum by 60% (0.60 ± 0.04 vs. 1.48 ± 0.13 mm, *P* < 0.0001, Fig. 6a) and by 28% for the D_2_ MSNs (0.78 ± 0.09 vs. 1.08 ± 0.08 mm, *P* = 0.0191, Fig. 6b). Surprisingly, no significant differences between sham and 6-OHDA-lesioned mice were observed regarding the total dendritic length of the D_1_/D_2_ MSNs (0.61 ± 0.05 vs. 0.75 ± 0.07 mm, *P* = 0.6356, Fig. 6c). Accordingly, in 6-OHDA-lesioned mice, a lower number of dendritic branching points were observed for the D_1_ MSNs (8.7 ± 0.7 vs. 16.6 ± 1.5 branching points, *P* < 0.0001) and the D_2_ MSNs (10.7 ± 0.9 vs. 15.0 ± 1.4 branching points, *P* = 0.0120) but not for the D_1_/D_2_ MSNs with 9.5 ± 0.8 branching points in both experimental groups (*P* > 0.9999).

### The spine density on D_1_/D_2_ MSN dendrites is reduced in 6-OHDA-lesioned mice

**Figure 7:**
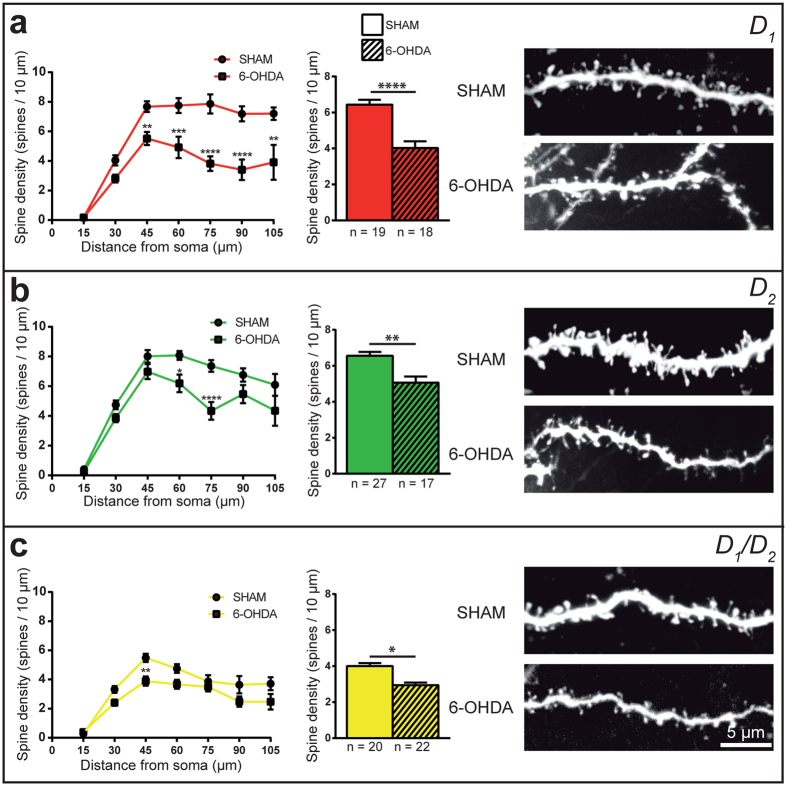
Dendritic spine density of the D_1_, D_2_ and D_1_/D_2_ MSNs in sham and 6-OHDA-lesioned mice. Sholl analysis of spine density (left column) and histograms showing the overall spine density (center column) of the D_1_ ((**a**), red), D_2_ ((**b**), green) and D_1_/D_2_ ((**c**), yellow) striatal MSNs in sham (circles and plain columns) and 6-OHDA (square and hatched columns) lesioned mice. The right column provides representative examples of dendritic segments belonging to the D_1_, D_2_ or D_1_/D_2_ MSNs that were filled with Lucifer yellow in sham and 6-OHDA-lesioned mice. *P < 0.05, **P < 0.01, ***P < 0.001, ****P < 0.0001 for sham vs. 6-OHDA by a Student’s T-test (histograms) or Two-way ANOVA followed by Bonferroni’s multiple comparison test (Sholl analysis).

Lesion of the striatal DA afferent projections leads to a significant decrease of spine density on dendrites belonging to the three types of MSNs. The overall spine density was 4.0 ± 0.4 spines/10 μm in 6-OHDA compared to 6.4 ± 0.3 in sham (*P* < 0.0001) for the D_1_ MSNs; 5.1 ± 0.3 spines/10 μm compared to 6.6 ± 0.2 (*P* = 0.0018) for the D_2_ MSNs and 3.0 ± 0.1 spines/10 μm compared to 4.0 ± 0.2 (*P* = 0.0427) for the D_1_/D_2_ MSNs. These reductions accounted for a 37.5% loss on the D_1_, 22.7% on the D_2_ and 25.0% on the D_1_/D_2_ MSNs. The Sholl analysis indicates that such lower spine density is maintained throughout the entire dendritic arborization of the three types of MSNs (Fig. 7).

## Discussion

The present study provides the first detailed description of the morphological characteristics, density and regional distribution of D_1_/D_2_ MSNs of the dorsal striatum and Acb in normal mice, as well as the first characterization of changes induced in this striatal subpopulation by striatal DA denervation. Our data gathered in normal animals reveal that the D_1_/D_2_ MSNs are morphologically distinct from the D_1_ and D_2_ MSNs: they have a smaller cell body, a less profusely arborized dendritic tree with branches that bear fewer spines than those of the D_1_ and D_2_ MSNs. They are uniformly scattered throughout the striatum, where they represent approximately 2% of the total number of MSNs, but heterogeneously distributed and more abundant in the Acb, where their proportion ranged from 7 to 15% of all MSNs. In 6-OHDA-lesioned mice, the density and regional distribution of all 3 types of MSNs is essentially unaltered. In contrast to the D_1_ and the D_2_ MSNs, the D_1_/D_2_ neurons do not show any significant reduction in the length of their dendritic arborization after intoxication with 6-OHDA. However, a reduction in dendritic spine density was noted in all three types of MSNs following DA depletion, but this pruning phenomenon was more prominent in the D_1_ than in the D_2_ or D_1_/D_2_ MSNs. The significance of these results will now be discussed in the light of relevant literature.

It is now well established that co-expression of D_1_ and D_2_ receptors occurs in some striatal MSNs, but there is still some controversy regarding the relative importance of such a unique neuronal population. In the literature, the percentage of striatal MSNs that coexpress D_1_ and D_2_ ranges from low^2^,^10^,^11^,^24^,^40^, moderate^12^,^14^,^41^ to nearly 100%^ ^9. Such substantial differences may be explained, on one hand, by the various methods and species used and, on the other hand, by the specificity of the antibodies or the *in situ* hybridization probes employed to detect D_1_ and D_2_ receptors. In the present study, depending on striatal sectors that were examined, we estimate that the D_1_/D_2_ MSNs account for 0.8–2.4% of the total number of striatal MSNs, a proportion that agrees with figures reported in other studies conducted in BAC transgenic mice^18^,^20^,^42^.

Despite that they represent only 2% of the total MSNs population of the adult mice dorsal striatum, the D_1_/D_2_ MSNs might play an important role in striatal functioning, as it is the case of striatal interneurons, which account only for 2–3% of striatal neurons in rodents^43^,^44^. Their presence throughout the dorsal striatum suggest that the D_1_/D_2_ MSNs are involved in the sensorimotor and associative functions of the striatum, which are integrated mainly within the caudolateral and the rostromedial sector of the structure, respectively^45^. However, the prevalence of the D_1_/D_2_ MSNs in the Acb indicates that these neurons are even more actively implied in the limbic aspect of striatal functioning. Indeed, we found the density of D_1_/D_2_ MSNs in the Acb to be significantly higher than in the dorsal striatum, a finding that is congruent with data from previous studies conducted in transgenic mice^20^,^21^,^24^,^46^,^47^ and by a higher number of D_1_/D_2_ heteromer in the rat and monkey Acb^25^,^26^. The shell compartment of the Acb was significantly more enriched in D_1_/D_2_ MSNs than the core compartment, supporting the notion that the latter is more similar to the dorsal striatum than the former, which has been described as a transition zone between the striatum and the extended amydgdala^48^. The fact that the D_1_/D_2_ MSNs form the vast majority of MSNs in the medial area of the shell compartment, as noted here, is interesting since this medial region of the Acb shell is known to be involved in feeding behaviors^49^,^50^,^51^,^52^ as well as in the response to noxious stimuli^52^. Whether or not the D_1_/D_2_ MSNs present in the Acb play a role in the antidepressant and anxiolytic effects observed after disruption of the D_1_-D_2_ complex^53^ remains to be investigated.

Besides their difference in the expression of DA receptors, the two major types of striatal MSNs express different neuropeptides in both rodents and primates^2^,^14^,^54^, the D_1_ MSNs containing SP and DYN while the D_2_ MSNs are enriched with ENK^20^. As for the D_1_/D_2_ MSNs, a previous report has suggested that they express both ENK and DYN in the rat^24^, whereas the present findings clearly show that they display immunoreactivity only for DYN. Such a discrepancy might reflect a species difference, but most likely results from a variation in the methodological approach. We used highly specific transgenic fluorescent reporters to identify the D_1_, D_2_ and D_1_/D_2_ striatal MSNs, whereas Perreault, *et al*.^24^ employed antibodies against the D_1_ and D_2_ receptors, which are known to be also highly expressed in the striatal neuropil, a situation that might have hampered the proper identification of the various peptide expressing MSNs. Our data indicate that, in regard to neuropeptide content, the striatal D_1_/D_2_ neurons in mice have more in common with the D_1_ than with the D_2_ MSNs.

This first detailed report on the morphological organization of D_1_/D_2_ MSNs reveals that these neurons have a smaller cell body and a shorter dendritic tree than their D_1_ or D_2_ counterparts. Our data also underline the less profuse dendritic aborization of the D_2_ compared to the D_1_ MSNs, a morphological feature that might explain, at least in part, why the D_2_ MSNs are more excitable than D_1_ MSNs^55^. Based on the morphology of their somatodendritic domains, it is tempting to speculate that the excitability and input resistance of the D_1_/D_2_ MSNs would be higher than the D_1_ or even the D_2_ MSNs, but such a view needs to be confirmed by detailed investigations of electrophysiological properties of the D_1_/D_2_ MSNs.

In addition to a smaller dendritic tree, the D_1_/D_2_ MSNs also contain less dendritic spines than D_1_ and D_2_ MSNs. The head of dendritic spines is the preferential synaptic contact site of glutamatergic corticostriatal projections arising from the cerebral cortex and the intralaminar thalamic nuclei^56^. With their less profuse dendritic arborization, the D_1_/D_2_ MSNs might receive significantly less glutamatergic input and thus be less vulnerable to excitotoxicity involved in different neuropathological conditions such as Huntington’s disease. More importantly in the context of the present study is the spatial distribution of DA terminals in contact with different parts of the MSNs. Those terminals that contact the cell body and proximal dendritic shafts might produce a relatively non-specific effect mediated by the volumic release of DA^57^. In contrast, as suggested Freund and colleagues^58^, the major DA input that occurs on the necks of dendritic spines is likely to be much more selective since it could prevent the excitatory glutamatergic input to the same spines from reaching the dendritic shaft. One of the main functions of striatal DA release might be to alter the pattern of firing of striatal output neurons by regulating their input^58^.

Lesions with 6-OHDA in rats were shown to increase the expression of D_2_ and decrease that of D_1_ by MSNs^59^. Here we report that the density and regional distribution of D_1_/D_2_ MSNs in mice striatum are unaltered by 6-OHDA lesions, suggesting that DA denervation does not alter the D_1_ expression by D_2_ MSNs or the D_2_ expression by D_1_ MSNs. However, it should be noted that the BAC transgenic reporter system used here does not fully allow to rule out the possibility that DA lesion may induce more subtle variations of DA receptor gene expression or receptor trafficking and degradation that could have remained undetected. The D_1_/D_2_ MSNs do not display any reduction of their dendritic tree following 6-OHDA lesions, in contrast to D_1_ and D_2_ MSNs, but the D_1_/D_2_ MSNs show a lower dendritic spine density in the DA-denervated striatum, as it is also the case for D_1_ and D_2_ MSNs. Reduction of the dendritic length of D_1_ and D_2_ MSNs following DA lesion is supported by observations gathered in mice^34^,^35^, monkeys^36^ and PD patients^37^,^38^,^39^. However, some studies in rats^60^ and mice^33^ failed to demonstrate such a phenomenon. The time between DA lesion and animal sacrifice might explain such a discrepancy. The interval between 6-OHDA injection and animal perfusion in the latter two studies ranged from 4 to 5 weeks, whereas the delay was much longer (8 weeks) in the present study. In face of such differences, it is tempting to speculate that the reduction in dendritic length occurs after the spine loss has occurred, that is in the late stages of the disease.

We documented spine loss for the three types of striatal MSNs, in accordance with data obtained for D_1_ and the D_2_ MSNs in mice^33^,^35^,^61^, rats^60^,^62^,^63^, monkeys^36^ and PD patients^37^,^38^. Interestingly, other studies have suggested that such spine loss may be restricted to the D_2_ MSNs in mice^31^,^32^. As mentioned above, DA axons are ideally positioned on the dendritic spine neck to influence the effect of corticostriatal and thalamostriatal glutamatergic input^56^. It has been hypothesized that the loss of DA afferents may destabilize the morphological integrity of the spine, potentially leading to spine pruning, a phenomenon that might be the result of maladaptive calcium influx through the L-type calcium channels located on MSNs^31^. Moreover, evidence has recently been gathered regarding the implication of acetylcholine, the level of which is known to be increased in PD^64^,^65^, in spine pruning of the D_2_ MSNs through the modulation of Kir2 channels, leading to an increase of dendritic excitability driven by the activation of M1 muscarinic receptor^32^.

The exact function of the D_1_/D_2_ MSNs of the striatum remains elusive. Colocalization of the D_1_ and D_2_ DA receptors has been reported in axons located in various basal ganglia components^24^. Whether this observation indicates a distinct striatofugal pathway remains unclear^66^. However, the existence of such unique striatofugal projections would imply a complementary functional role of the D_1_/D_2_ MSNs, working in concert with the D_1_ and the D_2_ MSNs for harmonious basal ganglia functioning. Single-axon tracing studies in rodents^6^,^67^ and monkeys^7^,^68^ have emphasized the highly collateralized nature of the striatofugal projections, challenging the concept of a simple dual (direct/indirect) striatofugal system. Whether the D_1_/D_2_ MSNs contribute to these highly collateralized striatofugal projections is currently unknown and single-axon tracing of D_1_/D_2_ MSNs is obviously needed to better appreciate their functional role in the basal ganglia functioning.

## Methods

### Animals

**Figure 8:**
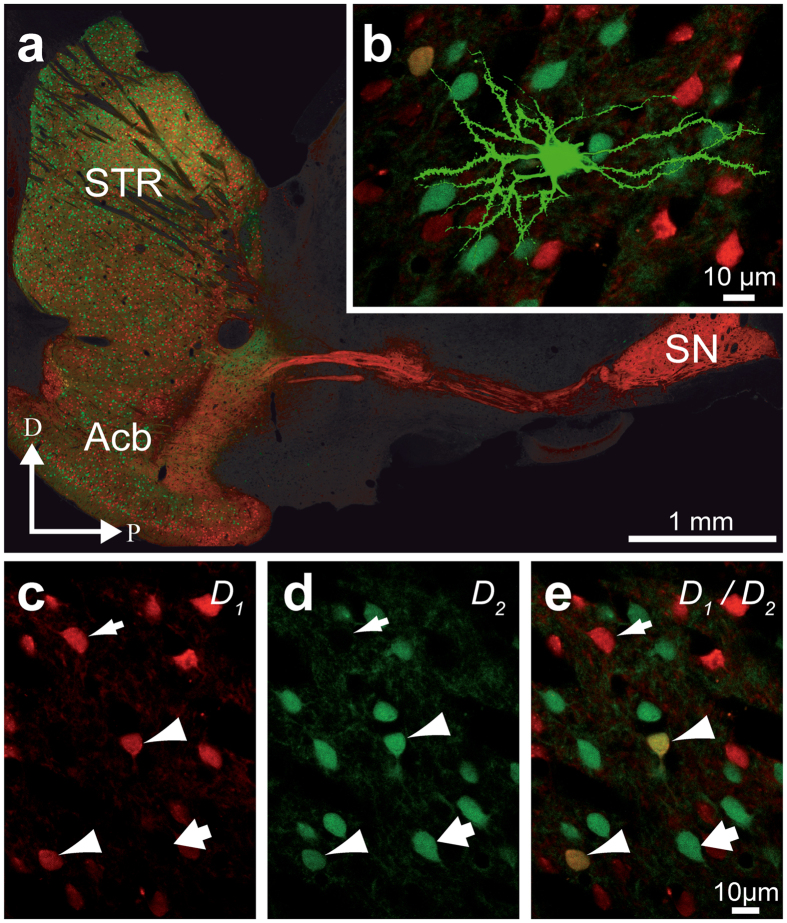
D_1_/D_2_ double BAC transgenic mice. Confocal images from the Drd1a-tdTomato/Drd2-EGFP double BAC transgenic mice (D_1_/D_2_) in which the expression of a red fluorescent protein (tdTomato) is under control of the D_1_ promoter and the expression of a green fluorescent protein (EGFP) is under control of the D_2_ promoter. (**a**) Confocal image of a sagittal section from a D_1_/D_2_ transgenic mouse taken through the striatum (STR) and the substantia nigra (SN). (**b)** Example of a Lucifer yellow-injected MSN located in the dorsal STR. (**c–e)** High magnification of striatal MSNs that contain the D_1_ (red, thin arrows), the D_2_ (green, thick arrows) or both D_1_/D_2_ (yellow, arrowheads) dopamine receptors.

This study was carried out on 25 double BAC transgenic mice (*Drd1a*-tdTomato/*Drd2*-EGFP) of 2 month old weighing between 20–30 g. Equal numbers of male and female were used. These D_1_/D_2_ mice were generated by breeding B6SJLF1-D_1_tdTomato BAC transgenic mice^19^ with C57Bl6J-D_2_-EGFP BAC animals^69^. They allow direct identification of the D_1_, D_2_ and D_1_/D_2_ MSNs (Fig. 8). In order to minimize over expression artifacts, all mice where heterozygous for each individual BAC transgene. Animals were housed under a 12 h light-dark cycle with water and food ad libitum. All procedures were approved by the *Comité de Protection des Animaux de l’Université Laval,* in accordance with the Canadian Council on Animal Care’s Guide to the Care and Use of Experimental Animals (Ed2) and with the ARRIVE guidelines. Maximum efforts were made to minimize the number of animals used.

### Stereotaxic injections

#### 6-OHDA unilateral injection and behavioral assessment

Nineteen *Drd1a*-tdTomato/*Drd2*-EGFP transgenic mice received an intracerebral injection of 6-OHDA (catalog no. H4381; Sigma-Aldrich, Saint-Louis, MO, USA) in the right medial forebrain bundle (mfb). Approximately 30 minutes before 6-OHDA administration, mice received intraperitoneal injection of desipramine (25 mg/kg) diluted in saline (0.9%) at a concentration of 2 mg/mL. Mice were then anaesthetized using 2% isoflurane and their head were fixed in a stereotaxic apparatus. A hole was drilled and the following stereotaxic coordinates were aimed: anteroposterior (bregma) = −1.2 mm; mediolateral = 1.1 mm; dorsoventral = −5.0 mm, corresponding to the mfb, according to the mouse brain atlas of Franklin and Paxinos^70^. A glass micropipette of 35 μm diameter at the tip containing a freshly prepared 6-OHDA solution was introduced in the mfb. The 6-OHDA was then pressure-injected and the micropipette was left in place for 2 min both prior and following the injection. The 6-OHDA was diluted in ascorbic acid (catalog no. A5960; Sigma-Aldrich) at a concentration of 6 μg/μL. A total volume of 0.25 μL of 6-OHDA, corresponding to a dose of 1.5 μg of the neurotoxin, was injected into the right mfb. The sham-lesioned group was composed of 4 mice that only received injections of the vehicle (0.02% ascorbic acid). After surgery, the skin was sutured and mice were allowed to recover.

Thirty days after surgery, mice from the two experimental groups were introduced in a large glass cylinder and spontaneous motor behavior was recorded during 10 min. Spontaneous rotations were counted by a blinded experimenter. Complete ipsilateral and contralateral rotations to the 6-OHDA-lesioned side were counted and used as behavioral indication of the severity of the DA lesion.

Fifty-six days after the 6-OHDA lesion, animals were deeply anesthetized with a mixture of ketamine (100 mg/kg) and xylazine (10 mg/kg). They were transcardially perfused with an initial wash of 40 mL of ice-cold sodium phosphate-buffered saline (PBS, 0.1 M; pH 7.4), followed by 150 mL of paraformaldehyde (PFA, 4% diluted in phosphate buffer). Brains were dissected out, post-fixed for 24 h in a 4% PFA solution and cut with a vibratome (model VT1200; Leica, Germany) into 50 μm-thick coronal sections, which were serially collected in sodium phosphate-buffered saline (PBS, 0.1 M, pH 7.4). The pre and post-commissural parts of the striatum were cut at 50 μm and used for immunohistochemistry and stereology whereas the commissural part was cut at 250 μm for intracellular injections.

#### Bilateral colchicine injections

Two other double BAC transgenic mice received bilateral injections of colchicine (catalog no. C9754; Sigma-Aldrich) in the striatum, a drug that block axonal transport, allowing optimal immunostaining of neuropeptides contained in MSNs cell bodies. For intra-cerebral injections of colchicine, the following stereotaxic coordinates were used: anteroposterior (bregma) = 0.14 mm; mediolateral = 2.00 mm; dorsoventral = 3.20 mm, corresponding to the dorsal part of the striatum at the commissural level, according to the mouse brain atlas of Franklin and Paxinos^70^. One μL of colchicine diluted at 23 mg/mL in saline was pressure-injected in each side of the brain. One week after injections, colchicine-injected mice were transcardially perfused, as described above. Their whole brains were dissected out and cut with a vibratome into 50 μm-thick transverse sections.

### Immunohistochemistry

#### TH and DAT immunohistochemistry

To assess the extent of the DA lesion induced by 6-OHDA injections, one 50 μm-thick section was selected from the pre-commissural striatum (0.14 mm from bregma) and from the SNc (−3.52 mm from bregma), in each mouse. These sections were immunostained for TH, the catalytic enzyme of DA synthesis, by using a polyclonal antibody (catalog no. AB152; Millipore Corporation, Billerica, USA) raised in rabbit. Briefly, the free-floating sections were sequentially incubated in (i) a blocking solution of PBS containing 2% normal goat serum and 0.01% Triton X-100 (1 h, RT); (ii) the same solution containing a 1/1000 dilution of rabbit polyclonal antibody against TH (overnight, 4 °C); and (iii) a 1/500 solution of biotinylated goat anti-rabbit (catalog no. BA-1000; Vector Laboratories, Burlingame, CA, USA) diluted in the same blocking solution (2 h at room temperature (RT)). After rinses in PBS, sections were incubated for 1 h at RT in an avidin-biotin-peroxydase complex solution (Vector Laboratories) diluted 1/100 in the blocking solution. Sections were then rinsed and the bound peroxidase revealed by incubating the sections for 3 min at RT in a 0.025% solution of 3,3′diaminobenzidine tetrahydrochloride (DAB; catalog no. D5637; Sigma-Aldrich) diluted in Tris-buffered saline (TBS; 50 mM; pH 7.4), to which 0.005% of H_2_O_2_ was added. The reaction was stopped and the sections mounted on gelatin-coated slide and air-dried. Sections were then dehydrated in graded alcohol, cleared in toluene and coverslipped with Permount (catalog no. SP15-500; Fisher Scientific). The TH-immunostained sections taken through the midbrain were used to assess the number of DA cell bodies in the SNc and VTA, as delineated based on the mouse brain atlas of Franklin et Paxinos^70^. On these sections, all TH+ cell bodies were counted and results expressed as percentage of control side.

In each mouse, the DA lesion was also assessed using an infrared imaging system (Odyssey CLx; LI-COR Biosciences, Lincoln, NE, USA) from a 50 μm-thick section taken at the pre-commissural level of the striatum (1.34 mm from bregma). Sections were immunostained for TH and DAT, using secondary antibodies coupled to infrared fluorescent dyes. The primary antibody against TH was the same as above (1/1000, overnight at 4 ^o^C). The monoclonal antibody against DAT (1/1000, overnight at 4 ^o^C, catalog no. MAB369; EMD Millipore Corporation, Billerica, USA) was raised in rat. Donkey anti-rabbit 680 (1/1000, 2 h at RT, catalog no. 926-68073; LI-COR Biosciences) and goat anti-rat 800 (1/1000, 2 h at RT, catalog no. 926-32219; LI-COR Biosciences) were used as secondary antibodies. Two solid-state diode lasers (685 nm and 785 nm) were used to excite secondary antibodies coupled to infrared fluorescent dyes. Intensity values of TH and DAT immunoreactivity were taken from six 0.16 mm^2^ squares randomly placed over the striatum and from one 0.16 mm^2^ square placed over the Acb.

#### Calbindin immunohistochemistry

In order to precisely delineate the core and the shell regions of the Acb, transverse sections adjacent to those used for stereology were immunostained for calbindin (CB). Briefly, the immunoperoxidase protocol described above was used but with a monoclonal primary antibody against CB (1/500, overnight at 4 ^o^C, catalog no. C-9848, Sigma-Aldrich) and a biotinylated horse anti-mouse secondary antibody (1/200, 2 h at RT, catalog no. BA-2000; Vector Laboratories).

#### μ-opioid receptor immunohistochemistry

In order to delineate the striosomes and matrix striatal compartments, 2 transverse sections were taken at the pre-commissural and post-commissural striatal levels and immunostained for μ-opioid receptor (MOR). These sections were used to assess differences in the density of D_1_/D_2_ MSNs between the 2 striatal compartments. Briefly, sections were incubated with a primary antibody against MOR (1/200, overnight at 4 ^o^C, catalog no. AB5509; Chemicon, Darmstadt, Germany) followed by incubation into a donkey anti-guinea pig secondary antibody coupled to Cy5 (1/200, 2 h at RT, catalog no. 706-175-148; Jackson ImmunoResearch, West Grove, PA, USA).

#### Enkephalin and dynorphin immunohistochemistry

Neuropeptide content of the D_1_, D_2_ and D_1_/D_2_ MSNs was assessed on singly labeled sections for ENK or DYN, taken from the 2 colchicine-injected mice. Briefly, sections were incubated with either a mouse anti-ENK (1/50, NOC1; Medicorp, Montreal, QC, Canada) or a rabbit anti-DYN (1/200, catalog no. SP1215; Acris, San Diego, CA, USA) antibody overnight at 4 ^o^C. Sections were then incubated with either a horse anti-mouse (1/200, catalog no. BA-2000; Vector Laboratories) biotinylated secondary antibodies for 2 h at RT, followed by streptavidin-Cy5 (1/200, catalog no. SA1011; Invitrogen, Carlsbad, CA, USA) for 1 h at RT in the case of ENK, or directly with a goat anti-rabbit-Cy5 (1/200, catalog no. 111-175-003; Jackson ImmunoResearch) for 2 h at RT in the case of DYN. Images were acquired using a confocal microscope (Zeiss, LSM 700; Oberkochen, Germany).

### Quantitative assessment of D_1_, D_2_ and D_1_/D_2_ MSNs in the striatum

Four sham and 4 6-OHDA-lesioned mice were used for stereological estimation of the number of D_1_, D_2_ and D_1_/D_2_ MSNs. In each mouse, 6 sections were selected from the pre-commissural striatum. Three adjacent sections were selected at 0.26 mm from bregma and 3 others at 1.10 mm. Three more sections were taken at −0.94 mm from bregma. These sections were used to estimate the number of D_1_, D_2_ and D_1_/D_2_ MSNs in the pre-commissural (0.26 mm from bregma) and post-commissural striatum (−0.94 mm from bregma), as well as in the Acb (1.10 mm from bregma), with an unbiased stereological method using a confocal microscope equipped with a digital camera and a motorized stage controlled by a computer running the StereoInvestigator software (v. 7.00.3; MicroBrightField, Colchester, VT, USA). First, on each section, striatal contour was traced and 4 striatal sectors were delineated corresponding to a dorsolateral, dorsomedial, ventrolateral and ventromedial sectors (Fig. 4). The Acb was divided into its core and shell compartments based on CB-immunostained adjacent sections.

On each section, the striatum was entirely scanned through multiple Z-stacks using a 40x objective (NA 1.4, oil immersion, Plan-Apochromat, Zeiss) and the 488 and 568 diode lasers at 2% of power. Optical resolution of the Z-stacks was 512 × 512 pixel (pixel size = 0.10 μm^2^), with Z-steps of 3 μm corresponding to the optical slice determined by the pinhole. The thickness of Z-stacks was fixed at 30 μm and the gain setting for each channel was kept constant during the entire acquisition process.

The process leading to the estimation of the total number of D_1_ and D_2_ MSNs began by randomly translating a grid formed by 235 × 580 μm rectangles on the previously acquired confocal images of the striatum and the Acb. At each intersection of the grid that fell into the sector, a counting frame measuring 157 × 157 μm was drawn and examined. Cell bodies containing tdTomato (D_1_) or GFP (D_2_) that fell inside the counting frame and did not contact the exclusion lines were counted whenever they came into focus within a 12 μm-thick optical disector placed at 9 μm from the top of the section. An average number of 2939 ± 186 D_1_ and 2477 ± 160 D_2_ neurons were counted in the striatum and 1187 ± 98 D_1_ and 642 ± 64 D_2_ cell bodies in the Acb of each mouse, yielding coefficient of error (Gundersen, m = 1) ranging between 0.05 and 0.18.

Because less numerous, the number of striatal cells containing both tdTomato (D_1_) and GFP (D_2_), here called the D_1_/D_2_ MSNs, was estimated with stereological parameters different from those employed to estimate the number of D_1_ and D_2_ MSNs. Selected transverse sections of the striatum were entirely examined with an optical disector of the same size of the one described above. An average of 599 ± 106 D_1_/D_2_ cells were counted in the striatum compared to 993 ± 191 D_1_/D_2_ neurons in the Acb of each mouse, yielding coefficient of error (Gundersen, m = 1) ranging between 0.08 and 0.27. The density of D_1_, D_2_ and D_1_/D_2_ MSNs was obtained by dividing the number of striatal cells estimated with the optical disector by the volume of striatal sectors sampled, as estimated with the Cavalieri’s method^71^.

In the 4 sham-lesioned animals, the 2 transverse sections taken at the pre-commissural (0.26 mm from bregma) and post-commissural (−0.94 mm from bregma) striatal levels that were immunostained for MOR were used to estimate the number of D_1_/D_2_ MSNs in the striosomes and matrix compartments. Again, all striatal D_1_/D_2_ MSNs were counted from previously acquired confocal images and the striosomes and matrix compartments were delineated by using MOR immunoreactivity.

### Single-cell injections of identified MSNs

Fine morphological changes of MSN dendritic arborization induced by DA denervation were characterized by single-neuronal injections of Lucifer yellow applied to PFA-fixed brain sections from the *Drd1a*-tdTomato/*Drd2*-EGFP transgenic mice, using a method previously described^72^,^73^. Sharp heat-pulled glass micropipettes filled with a 4% solution of Lucifer yellow (catalog no. L453; Life Technologies, Carlsbad, CA, USA) and containing a silver electrode connected to a computer-controlled microelectrode amplifier (Multiclamp 700 A, Axon Instruments) were inserted into 250 μm-thick brain section kept in ice-cold PBS (0.1 M), under an epifluorescence microscope (model no. E600FM, Nikon, Tokyo). After the insertion of the micropipette into the soma of visually identified MSNs located in the dorsal striatum, a negative direct current of 5 nA was administered for 20 minutes during which MSNs were filled with the negatively charged Lucifer yellow tracer. A 488 nm filter was used to visualize GFP contained in D_2_ MSNs whereas a 568 nm filter was used to detect the presence of tdTomato into D_1_ MSNs. Each neurons being injected was carefully inspected with both filters in order to determine content in GFP and/or tdTomato with a 40X water immersion objective (NA 0.80). All injected MSNs were located in the dorsal striatal region, at the commissural level, and restricted to 0.02–0.26 mm in the anteroposterior axis relative to the bregma, 1–3 mm in the mediolateral axis and 2–3 mm in the dorsoventral axis, according to the stereotaxic mouse brain atlas^70^.

The 250 μm-tick sections containing injected striatal neurons were mounted on glass slides and coverslipped with a fluorescence mounting medium (S3023; Dako, Mississauga, Ontario, Canada). Z-stack of Lucifer yellow-filled neurons were obtained from the confocal microscope using a 405 nm diode laser and a 63x oil immersion objective (NA 1.4, Plan-Apochromat, Zeiss). The pixel size was 0.001 μm^2^ whereas the optical slicing was 0.3 μm. A tiling process was used when dendritic arborization extend beyond the field of view.

### Morphological analysis of injected MSNs

Analyses of dendritic arborization and spine density were performed using the freely available *NeuronStudio* software (CNIC, Mount Sinai School of Medecine, New York, NY, USA). The entire somatodendritic domains of injected neurons were carefully reconstructed using maximum intensity projection in *NeuronStudio* software, as previously described^74^. By using a combination of automatic spine detection from *NeuronStudio* software followed by a careful examination of individual spine, we were able to provide faithful three-dimensional reconstructions of somatodendritic domains of injected MSNs. From these reconstructions, Sholl analyses were performed on individual MSNs of the D_1_, D_2_ and D_1_/D_2_ types. Measurements of the size of the cell body were conducted by using the *ImageJ* software (Version 1.48). In this software, maximum intensity projections of Z-stacks were generated and diameters of cell bodies were measured. Dendritic arborization analyses and size measurements were performed on 19 D_1_, 27 D_2_, 20 D_1_/D_2_ Lucifer-yellow-injected MSNs from 4 sham-lesioned animals and on 18 D_1_, 17 D_2_ and 22 D_1_/D_2_ MSNs from 19 6-OHDA-lesioned mice.

### Statistical analysis

Statistical differences of neuronal densities between different striatal and Acb regions were assessed using Kruskal-Wallis non-parametric statistical test. Differences between sham and 6-OHDA-lesioned mice regarding neuronal densities and spontaneous rotations were assessed with Mann-Whitney non-parametric test. Variations in immunoreactivity for TH and DAT between sham and 6-OHDA-lesioned mice were detected using the Mann-Whitney statistical test. A Sholl analysis with 15 μm increments was used to determine the spine density and statistical differences for dendritic morphology (number of branching points, spine density and dendritic length) and for cell body diameters between the two experimental groups and the three types of MSNs were assessed with the two-way analysis of variance (ANOVA) followed by a Bonferroni multiple comparison test. All statistical tests were performed using GraphPad Prism software (v. 6.01; GraphPad Software, San Diego, CA, USA). Mean and standard error of the mean are used throughout the text as central tendency and dispersion measure, respectively.

## Additional Information

**How to cite this article**: Gagnon, D. *et al*. Striatal Neurons Expressing D_1_ and D_2_ Receptors are Morphologically Distinct and Differently Affected by Dopamine Denervation in Mice. *Sci. Rep.* **7**, 41432; doi: 10.1038/srep41432 (2017).

**Publisher's note:** Springer Nature remains neutral with regard to jurisdictional claims in published maps and institutional affiliations.

## Supplementary Material

Supplementary Figure S1

## References

[b1] Graveland G. A. & DiFiglia M. The frequency and distribution of medium-sized neurons with indented nuclei in the primate and rodent neostriatum. Brain Res 327, 307–311 (1985). 3986508 10.1016/0006-8993(85)91524-0

[b2] Gerfen C. R. . D1 and D2 dopamine receptor-regulated gene expression of striatonigral and striatopallidal neurons. Science 250, 1429–1432 (1990). 2147780 10.1126/science.2147780

[b3] Le Moine C. & Bloch B. D1 and D2 dopamine receptor gene expression in the rat striatum: sensitive cRNA probes demonstrate prominent segregation of D1 and D2 mRNAs in distinct neuronal populations of the dorsal and ventral striatum. The Journal of comparative neurology 355, 418–426 (1995). 7636023 10.1002/cne.903550308

[b4] Wichmann T. & DeLong M. R. Models of basal ganglia function and pathophysiology of movement disorders. Neurosurgery clinics of North America 9, 223–236 (1998). 9495888

[b5] Albin R. L., Young A. B. & Penney J. B. The functional anatomy of disorders of the basal ganglia. Trends in neurosciences 18, 63–64 (1995). 7537410

[b6] Wu Y., Richard S. & Parent A. The organization of the striatal output system: a single-cell juxtacellular labeling study in the rat. Neuroscience research 38, 49–62 (2000). 10997578 10.1016/s0168-0102(00)00140-1

[b7] Lévesque M. & Parent A. The striatofugal fiber system in primates: a reevaluation of its organization based on single-axon tracing studies. Proceedings of the National Academy of Sciences of the United States of America 102, 11888–11893 (2005). 16087877 10.1073/pnas.0502710102 PMC1187973

[b8] Kupchik Y. M. . Coding the direct/indirect pathways by D1 and D2 receptors is not valid for accumbens projections. Nature neuroscience 18, 1230–1232 (2015). 26214370 10.1038/nn.4068 PMC4551610

[b9] Aizman O. . Anatomical and physiological evidence for D1 and D2 dopamine receptor colocalization in neostriatal neurons. Nature neuroscience 3, 226–230 (2000). 10700253 10.1038/72929

[b10] Aubert I., Ghorayeb I., Normand E. & Bloch B. Phenotypical characterization of the neurons expressing the D1 and D2 dopamine receptors in the monkey striatum. The Journal of comparative neurology 418, 22–32 (2000). 10701753

[b11] Lester J., Fink S., Aronin N. & DiFiglia M. Colocalization of D1 and D2 dopamine receptor mRNAs in striatal neurons. Brain Res 621, 106–110 (1993). 8221060 10.1016/0006-8993(93)90303-5

[b12] Meador-Woodruff J. H. . Comparison of the distributions of D1 and D2 dopamine receptor mRNAs in rat brain. Neuropsychopharmacology: official publication of the American College of Neuropsychopharmacology 5, 231–242 (1991). 1839499

[b13] Shetreat M. E., Lin L., Wong A. C. & Rayport S. Visualization of D1 dopamine receptors on living nucleus accumbens neurons and their colocalization with D2 receptors. Journal of neurochemistry 66, 1475–1482 (1996). 8627301 10.1046/j.1471-4159.1996.66041475.x

[b14] Surmeier D. J., Song W. J. & Yan Z. Coordinated expression of dopamine receptors in neostriatal medium spiny neurons. The Journal of neuroscience: the official journal of the Society for Neuroscience 16, 6579–6591 (1996). 8815934 10.1523/JNEUROSCI.16-20-06579.1996 PMC6578920

[b15] Surmeier D. J., Yan Z. & Song W. J. Coordinated expression of dopamine receptors in neostriatal medium spiny neurons. Advances in pharmacology 42, 1020–1023 (1998). 9328072 10.1016/s1054-3589(08)60921-7

[b16] Weiner D. M. . D1 and D2 dopamine receptor mRNA in rat brain. Proceedings of the National Academy of Sciences of the United States of America 88, 1859–1863 (1991). 1825729 10.1073/pnas.88.5.1859 PMC51125

[b17] Yung K. K. . Immunocytochemical localization of D1 and D2 dopamine receptors in the basal ganglia of the rat: light and electron microscopy. Neuroscience 65, 709–730 (1995). 7609871 10.1016/0306-4522(94)00536-e

[b18] Thibault D., Loustalot F., Fortin G. M., Bourque M. J. & Trudeau L. E. Evaluation of D1 and D2 dopamine receptor segregation in the developing striatum using BAC transgenic mice. PloS one 8, e67219 (2013). 23843993 10.1371/journal.pone.0067219 PMC3699584

[b19] Shuen J. A., Chen M., Gloss B. & Calakos N. Drd1a-tdTomato BAC transgenic mice for simultaneous visualization of medium spiny neurons in the direct and indirect pathways of the basal ganglia. The Journal of neuroscience: the official journal of the Society for Neuroscience 28, 2681–2685 (2008). 18337395 10.1523/JNEUROSCI.5492-07.2008 PMC6670676

[b20] Bertran-Gonzalez J. . Opposing patterns of signaling activation in dopamine D1 and D2 receptor-expressing striatal neurons in response to cocaine and haloperidol. The Journal of neuroscience: the official journal of the Society for Neuroscience 28, 5671–5685 (2008). 18509028 10.1523/JNEUROSCI.1039-08.2008 PMC6670792

[b21] Gangarossa G. . Spatial distribution of D1R- and D2R-expressing medium-sized spiny neurons differs along the rostro-caudal axis of the mouse dorsal striatum. Frontiers in neural circuits 7, 124 (2013). 23908605 10.3389/fncir.2013.00124 PMC3725430

[b22] Lee S. P. . Dopamine D1 and D2 receptor Co-activation generates a novel phospholipase C-mediated calcium signal. The Journal of biological chemistry 279, 35671–35678 (2004). 15159403 10.1074/jbc.M401923200

[b23] Perreault M. L., Fan T., Alijaniaram M., O’Dowd B. F. & George S. R. Dopamine D1-D2 receptor heteromer in dual phenotype GABA/glutamate-coexpressing striatal medium spiny neurons: regulation of BDNF, GAD67 and VGLUT1/2. PloS one 7, e33348 (2012). 22428025 10.1371/journal.pone.0033348 PMC3299775

[b24] Perreault M. L. . The dopamine D1-D2 receptor heteromer localizes in dynorphin/enkephalin neurons: increased high affinity state following amphetamine and in schizophrenia. The Journal of biological chemistry 285, 36625–36634 (2010). 20864528 10.1074/jbc.M110.159954 PMC2978591

[b25] Perreault M. L. . Disruption of a dopamine receptor complex amplifies the actions of cocaine. European neuropsychopharmacology: the journal of the European College of Neuropsychopharmacology 26, 1366–1377 (2016). 27480020 10.1016/j.euroneuro.2016.07.008

[b26] Rico A. J. . Neurochemical evidence supporting dopamine D1-D2 receptor heteromers in the striatum of the long-tailed macaque: changes following dopaminergic manipulation. Brain structure & function (2016). 10.1007/s00429-016-1306-x PMC5406426 27612857

[b27] Beaulieu J. M. & Gainetdinov R. R. The physiology, signaling, and pharmacology of dopamine receptors. Pharmacological reviews 63, 182–217 (2011). 21303898 10.1124/pr.110.002642

[b28] Beaulieu J. M., Espinoza S. & Gainetdinov R. R. Dopamine receptors - IUPHAR Review 13. British journal of pharmacology 172, 1–23 (2015). 25671228 10.1111/bph.12906 PMC4280963

[b29] Frederick A. L. . Evidence against dopamine D1/D2 receptor heteromers. Molecular psychiatry 20, 1373–1385 (2015). 25560761 10.1038/mp.2014.166 PMC4492915

[b30] Biezonski D. K., Trifilieff P., Meszaros J., Javitch J. A. & Kellendonk C. Evidence for limited D1 and D2 receptor coexpression and colocalization within the dorsal striatum of the neonatal mouse. The Journal of comparative neurology 523, 1175–1189 (2015). 25556545 10.1002/cne.23730 PMC4390438

[b31] Day M. . Selective elimination of glutamatergic synapses on striatopallidal neurons in Parkinson disease models. Nature neuroscience 9, 251–259 (2006). 16415865 10.1038/nn1632

[b32] Shen W. . Cholinergic modulation of Kir2 channels selectively elevates dendritic excitability in striatopallidal neurons. Nature neuroscience 10, 1458–1466 (2007). 17906621 10.1038/nn1972

[b33] Suarez L. M. . L-DOPA treatment selectively restores spine density in dopamine receptor D2-expressing projection neurons in dyskinetic mice. Biological psychiatry 75, 711–722 (2014). 23769604 10.1016/j.biopsych.2013.05.006

[b34] Toy W. A. . Treadmill exercise reverses dendritic spine loss in direct and indirect striatal medium spiny neurons in the 1-methyl-4-phenyl-1,2,3,6-tetrahydropyridine (MPTP) mouse model of Parkinson’s disease. Neurobiology of disease 63, 201–209 (2014). 24316165 10.1016/j.nbd.2013.11.017 PMC3940446

[b35] Fieblinger T. . Cell type-specific plasticity of striatal projection neurons in parkinsonism and L-DOPA-induced dyskinesia. Nature communications 5, 5316 (2014). 10.1038/ncomms6316 PMC4431763 25360704

[b36] Villalba R. M., Lee H. & Smith Y. Dopaminergic denervation and spine loss in the striatum of MPTP-treated monkeys. Experimental neurology 215, 220–227 (2009). 18977221 10.1016/j.expneurol.2008.09.025 PMC2680135

[b37] Zaja-Milatovic S. . Dendritic degeneration in neostriatal medium spiny neurons in Parkinson disease. Neurology 64, 545–547 (2005). 15699393 10.1212/01.WNL.0000150591.33787.A4

[b38] Stephens B. . Evidence of a breakdown of corticostriatal connections in Parkinson’s disease. Neuroscience 132, 741–754 (2005). 15837135 10.1016/j.neuroscience.2005.01.007

[b39] McNeill T. H., Brown S. A., Rafols J. A. & Shoulson I. Atrophy of medium spiny I striatal dendrites in advanced Parkinson’s disease. Brain Res 455, 148–152 (1988). 3416180 10.1016/0006-8993(88)90124-2

[b40] Deng Y. P., Lei W. L. & Reiner A. Differential perikaryal localization in rats of D1 and D2 dopamine receptors on striatal projection neuron types identified by retrograde labeling. Journal of chemical neuroanatomy 32, 101–116 (2006). 16914290 10.1016/j.jchemneu.2006.07.001

[b41] Ariano M. A., Stromski C. J., Smyk-Randall E. M. & Sibley D. R. D2 dopamine receptor localization on striatonigral neurons. Neuroscience letters 144, 215–220 (1992). 1436705 10.1016/0304-3940(92)90753-t

[b42] Escande M. V., Taravini I. R., Zold C. L., Belforte J. E. & Murer M. G. Loss of Homeostasis in the Direct Pathway in a Mouse Model of Asymptomatic Parkinson’s Disease. The Journal of neuroscience: the official journal of the Society for Neuroscience 36, 5686–5698 (2016). 27225760 10.1523/JNEUROSCI.0492-15.2016 PMC6601837

[b43] Tepper J. M. & Bolam J. P. Functional diversity and specificity of neostriatal interneurons. Current opinion in neurobiology 14, 685–692 (2004). 15582369 10.1016/j.conb.2004.10.003

[b44] Rymar V. V., Sasseville R., Luk K. C. & Sadikot A. F. Neurogenesis and stereological morphometry of calretinin-immunoreactive GABAergic interneurons of the neostriatum. The Journal of comparative neurology 469, 325–339 (2004). 14730585 10.1002/cne.11008

[b45] Parent A. & Hazrati L. N. Functional anatomy of the basal ganglia. I. The cortico-basal ganglia-thalamo-cortical loop. Brain research. Brain research reviews 20, 91–127 (1995). 7711769 10.1016/0165-0173(94)00007-c

[b46] Gangarossa G. . Distribution and compartmental organization of GABAergic medium-sized spiny neurons in the mouse nucleus accumbens. Frontiers in neural circuits 7, 22 (2013). 23423476 10.3389/fncir.2013.00022 PMC3575607

[b47] Hasbi A. . Calcium signaling cascade links dopamine D1-D2 receptor heteromer to striatal BDNF production and neuronal growth. Proceedings of the National Academy of Sciences of the United States of America 106, 21377–21382 (2009). 19948956 10.1073/pnas.0903676106 PMC2795506

[b48] Harlan R. E., Guillot M. & Garcia M. M. In The Basal Ganglia VI Vol. 54 (eds Graybiel A. M., DeLong M. R. & Kitai S. T.) 393–397 (Springer: US, 2003).

[b49] Kelley A. E. & Swanson C. J. Feeding induced by blockade of AMPA and kainate receptors within the ventral striatum: a microinfusion mapping study. Behavioural brain research 89, 107–113 (1997). 9475619 10.1016/s0166-4328(97)00054-5

[b50] Stratford T. R. & Kelley A. E. GABA in the nucleus accumbens shell participates in the central regulation of feeding behavior. The Journal of neuroscience: the official journal of the Society for Neuroscience 17, 4434–4440 (1997). 9151760 10.1523/JNEUROSCI.17-11-04434.1997 PMC6573549

[b51] Basso A. M. & Kelley A. E. Feeding induced by GABA(A) receptor stimulation within the nucleus accumbens shell: regional mapping and characterization of macronutrient and taste preference. Behavioral neuroscience 113, 324–336 (1999). 10357457 10.1037//0735-7044.113.2.324

[b52] Lopes A. P. . GABAA and GABAB agonist microinjections into medial accumbens shell increase feeding and induce anxiolysis in an animal model of anxiety. Behavioural brain research 184, 142–149 (2007). 17714798 10.1016/j.bbr.2007.07.001

[b53] Shen M. Y. . Rapid anti-depressant and anxiolytic actions following dopamine D1-D2 receptor heteromer inactivation. European neuropsychopharmacology: the journal of the European College of Neuropsychopharmacology 25, 2437–2448 (2015). 26431907 10.1016/j.euroneuro.2015.09.004

[b54] Steiner H. & Gerfen C. R. Role of dynorphin and enkephalin in the regulation of striatal output pathways and behavior. Experimental brain research 123, 60–76 (1998). 9835393 10.1007/s002210050545

[b55] Gertler T. S., Chan C. S. & Surmeier D. J. Dichotomous anatomical properties of adult striatal medium spiny neurons. The Journal of neuroscience: the official journal of the Society for Neuroscience 28, 10814–10824 (2008). 18945889 10.1523/JNEUROSCI.2660-08.2008 PMC3235748

[b56] Moss J. & Bolam J. P. A dopaminergic axon lattice in the striatum and its relationship with cortical and thalamic terminals. The Journal of neuroscience: the official journal of the Society for Neuroscience 28, 11221–11230 (2008). 18971464 10.1523/JNEUROSCI.2780-08.2008 PMC6671499

[b57] Descarries L. & Mechawar N. Ultrastructural evidence for diffuse transmission by monoamine and acetylcholine neurons of the central nervous system. Progress in brain research 125, 27–47 (2000). 11098652 10.1016/S0079-6123(00)25005-X

[b58] Freund T. F., Powell J. F. & Smith A. D. Tyrosine hydroxylase-immunoreactive boutons in synaptic contact with identified striatonigral neurons, with particular reference to dendritic spines. Neuroscience 13, 1189–1215 (1984). 6152036 10.1016/0306-4522(84)90294-x

[b59] Gerfen C. R. Molecular effects of dopamine on striatal-projection pathways. Trends in neurosciences 23, S64–70 (2000). 11052222 10.1016/s1471-1931(00)00019-7

[b60] Zhang Y. . Aberrant restoration of spines and their synapses in L-DOPA-induced dyskinesia: involvement of corticostriatal but not thalamostriatal synapses. The Journal of neuroscience: the official journal of the Society for Neuroscience 33, 11655–11667 (2013). 23843533 10.1523/JNEUROSCI.0288-13.2013 PMC3724545

[b61] Fieblinger T. & Cenci M. A. Zooming in on the small: the plasticity of striatal dendritic spines in L-DOPA-induced dyskinesia. Movement disorders: official journal of the Movement Disorder Society 30, 484–493 (2015). 25759263 10.1002/mds.26139

[b62] Nishijima H. . Morphologic changes of dendritic spines of striatal neurons in the levodopa-induced dyskinesia model. Movement disorders: official journal of the Movement Disorder Society 29, 336–343 (2014). 24573720 10.1002/mds.25826

[b63] Ingham C. A., Hood S. H., van Maldegem B., Weenink A. & Arbuthnott G. W. Morphological changes in the rat neostriatum after unilateral 6-hydroxydopamine injections into the nigrostriatal pathway. Experimental brain research 93, 17–27 (1993). 7682182 10.1007/BF00227776

[b64] Barbeau A. The pathogenesis of Parkinson’s disease: a new hypothesis. Canadian Medical Association journal 87, 802–807 (1962). 13966498 PMC1849683

[b65] Aosaki T., Miura M., Suzuki T., Nishimura K. & Masuda M. Acetylcholine-dopamine balance hypothesis in the striatum: an update. Geriatrics & gerontology international 10 Suppl 1, S148–157 (2010). 20590830 10.1111/j.1447-0594.2010.00588.x

[b66] Perreault M. L., Hasbi A., O’Dowd B. F. & George S. R. The dopamine d1-d2 receptor heteromer in striatal medium spiny neurons: evidence for a third distinct neuronal pathway in Basal Ganglia. Frontiers in neuroanatomy 5, 31 (2011). 21747759 10.3389/fnana.2011.00031 PMC3130461

[b67] Kawaguchi Y., Wilson C. J. & Emson P. C. Projection subtypes of rat neostriatal matrix cells revealed by intracellular injection of biocytin. The Journal of neuroscience: the official journal of the Society for Neuroscience 10, 3421–3438 (1990). 1698947 10.1523/JNEUROSCI.10-10-03421.1990 PMC6570194

[b68] Parent A., Charara A. & Pinault D. Single striatofugal axons arborizing in both pallidal segments and in the substantia nigra in primates. Brain Res 698, 280–284 (1995). 8581498 10.1016/0006-8993(95)01017-p

[b69] Gong S. . A gene expression atlas of the central nervous system based on bacterial artificial chromosomes. Nature 425, 917–925 (2003). 14586460 10.1038/nature02033

[b70] Franklin K. B. J. & Paxinos G. The mouse brain in stereotaxic coordinates. (Academic Press, 1997).

[b71] Gundersen H. J. & Jensen E. B. The efficiency of systematic sampling in stereology and its prediction. Journal of microscopy 147, 229–263 (1987). 3430576 10.1111/j.1365-2818.1987.tb02837.x

[b72] Buhl E. H. & Lubke J. Intracellular lucifer yellow injection in fixed brain slices combined with retrograde tracing, light and electron microscopy. Neuroscience 28, 3–16 (1989). 2668782 10.1016/0306-4522(89)90227-3

[b73] Dumitriu D., Rodriguez A. & Morrison J. H. High-throughput, detailed, cell-specific neuroanatomy of dendritic spines using microinjection and confocal microscopy. Nature protocols 6, 1391–1411 (2011). 21886104 10.1038/nprot.2011.389 PMC3566769

[b74] Bories C., Husson Z., Guitton M. J. & De Koninck Y. Differential balance of prefrontal synaptic activity in successful versus unsuccessful cognitive aging. The Journal of neuroscience: the official journal of the Society for Neuroscience 33, 1344–1356 (2013). 23345211 10.1523/JNEUROSCI.3258-12.2013 PMC6618737

