# Supplementary material for: Striatal Neurons Expressing D1 and D2 Receptors are Morphologically Distinct and Differently Affected by Dopamine Denervation in Mice
Source: Sci Rep. 2017 Jan 27;7:41432. doi: 10.1038/srep41432 (PMC5269744; doi:10.1038/srep41432)
Supplement: Supplementary Figure S1 [file srep41432-s1.pdf]

STRIATAL NEURONS EXPRESSING D_1 AND D_2 RECEPTORS ARE MORPHOGICALLY DISTINCT AND DIFFERENTLY AFFECTED BY DOPAMINE DENERVATION IN MICE

GAGNON D, PETRYSZYN S, SANCHEZ MG, BORIES C, BEAULIEU JM, DE KONINCK Y, PARENT A, PARENT M*

Supplementary Figure S1

D_1/D_2 MSN distribution in the striosomes and matrix striatal compartments

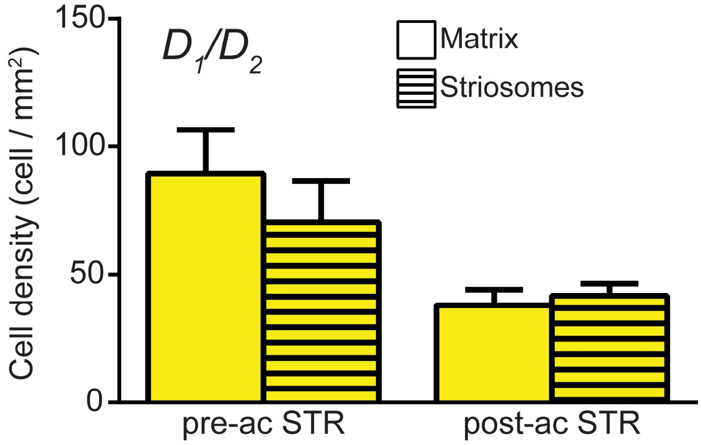

Supplementary Figure S1: ***D_1/D_2 MSN distribution in the striosomes and matrix striatal compartments.*** Histogram showing the density of D_1/D_2 MSNs observed in the matrix (plain columns) and striosomes (hatched columns) striatal compartments, at the pre-commissural and post-commissural levels.
